# CRISPR–Cas system to discover host-virus interactions in Flaviviridae

**DOI:** 10.1186/s12985-023-02216-7

**Published:** 2023-10-27

**Authors:** Zahra Ramezannia, Ali Shamekh, Hossein Bannazadeh Baghi

**Affiliations:** 1 Department of Virology, Faculty of Medicine, Tabriz University of Medical Sciences, Tabriz, Iran; 2 Department of Medical Virology, Faculty of Medicine, Hamadan University of Medical Sciences, Hamadan, Iran; 3 Infectious and Tropical Diseases Research Center, Tabriz University of Medical Sciences, Tabriz, 5166/15731 Iran; 4 Immunology Research Center, Tabriz University of Medical Sciences, Tabriz, Iran

**Keywords:** Flaviviridae pathogenesis, CRISPR/Cas, Antiviral strategy, Flavivirus, Host factors, Genome editing, CRISPR KO, Virus-host interactions, Genome-wide CRISPR screens

## Abstract

The Flaviviridae virus family members cause severe human diseases and are responsible for considerable mortality and morbidity worldwide. Therefore, researchers have conducted genetic screens to enhance insight into viral dependency and develop potential anti-viral strategies to treat and prevent these infections. The host factors identified by the clustered regularly interspaced short palindromic repeats (CRISPR) system can be potential targets for drug development. Meanwhile, CRISPR technology can be efficiently used to treat viral diseases as it targets both DNA and RNA. This paper discusses the host factors related to the life cycle of viruses of this family that were recently discovered using the CRISPR system. It also explores the role of immune factors and recent advances in gene editing in treating flavivirus-related diseases. The ever-increasing advancements of this technology may promise new therapeutic approaches with unique capabilities, surpassing the traditional methods of drug production and treatment.

## Introduction

The Flaviviridae family encompasses a large group of single-stranded, positive-sense RNA viruses. Four genera belong to this family: Flavivirus, Pestivirus, Hepacivirus, and Pegivirus. Some members of the Hepacivirus and Flavivirus genera are responsible for several important human diseases [1]. Hepatitis C virus (HCV) belongs to the Hepacivirus genus, which differs in many aspects compared to the members of the Flavivirus genus, including the transmission route or the course of infection [2]. The main transmission path for most flaviviruses is through arthropod vectors and includes important pathogens such as the Zika virus (ZIKV) [3]. Zika virus infections in pregnant women have been associated with congenital microcephaly and other developmental defects in infants. The aforementioned traits of Zika virus has attracted the attention of the medical community worldwide [4–6]. Dengue virus (DENV), which causes approximately 100 million symptomatic infections annually, is another cause of infectious diseases inflicted by flaviviruses [7]. Yellow fever virus (YFV) is another member of the Flavivirus genus and is known to be a cause of hemorrhagic fever. It remains prevalent in sub-Saharan Africa and South America, in spite of the availability of a highly effective live-attenuated vaccine against it [8, 9]. Among people infected with West Nile virus (WNV), only about 20% present the symptoms of West Nile fever (WNF). Less than 1% of the infected individuals develop a neuroinvasive disease characterized by encephalitis, meningitis, and flaccid paralysis [10–12]. So far, no specific or potent antiviral treatments are available against ZIKV, DENV, and WNV infections. Outbreaks still occur despite licensed vaccines against several members of the Flaviviridae family, including DENV, YFV, Japanese encephalitis virus (JEV), and Tick-borne encephalitis virus (TBEV), emphasizing the challenges and flaws in implementing effective vaccination programs [13]. Viruses are obligate pathogens, dependent on their host to complete their replication cycle. Viruses utilize cellular receptors to enter the host and hijack cellular functions and pathways to replicate, assemble and release new virus particles; hence, identifying the cellular factors that promote or restrict virus replication will reveal the fundamental characteristics of host-virus interaction. This, in turn, could lead to the development of target-specific antiviral drugs in the future [14]. Genomic approaches are increasingly being utilized to identify viral pathogenesis mechanisms and study host-viral interactions. Several genetic screening technologies, such as RNA interference (RNAi), haploid embryonic stem cells, and clustered regularly interspaced short palindromic repeats (CRISPR), have proven to be powerful means for examining viral lifecycles [15]. However, the CRISPR/Cas technology as an efficient tool for genomic engineering has overcome the limitations of other competing technologies. Furthermore, this system has been engineered to effectively induce knockout mutations in a wide range of cell types. The expansion of CRISPR/Cas9 screening libraries allows all known genes from any species to be targeted, including a pool of guide RNAs to target a vast variety of genes. Either way, gene knockouts or the activation of gene expression can be achieved [16, 17]. The present review aims to elucidate the basic principles and types of different CRISPR screens, and their use in novel anti-viral approaches. Therefore, we have also provided a comprehensive overview of the recent discoveries about virus-host interactions which have been achieved using CRISPR screens. Lastly, we have described the currently available CRISPR-Cas antiviral strategies against the Flaviviridae family as one of the main groups of lethal viral infections.

## CRISPR/Cas system

CRISPR/Cas system is an adaptive immune mechanism protecting the bacteria against invading viruses and plasmids [18, 19]. Bacterial CRISPR loci consist of a Cas operon and a repeat-spacer array. This defensive process can be divided into three stages. The acquisition step involves the integration of foreign nucleic acids into a CRISPR array as new CRISPR spacers separated by repeat sequences found adjacent to the CRISPR-associated (Cas) genes, which encode Cas ribonucleases. This step creates a memory of the foreign genetic components [20]. In the second step (expression), the CRISPR array must be transcribed into a pre-CRISPR RNA transcript (pre-crRNA), then processed. The outcome of this step at this stage is finally a mature crRNA [21, 22]. Additionally, a transactivating RNA (tracrRNA) is also encoded by the CRISPR locus, which has complementarity properties to the repeat areas of crRNA transcripts [21]. Subsequently, in the third stage (i.e. interference), through the binding of complementary repeat region sequences, the crRNA-tracrRNA hybrid is formed. Finally, Cas nuclease is guided to the complementary DNA sequences using this RNA hybrid, targeting and cleaving the nucleic acids derived from the invading viruses and other genetic elements [23].

The CRISPR/Cas systems are divided into two categories according to their effector molecules: multi-subunit effector molecules in Class 1 and a single effector molecule in Class 2. The first class can be subdivided into three types (I, III, and IV), and Class 2 consists of types II, V, and VI [24, 25]. Specifically, the CRISPR/Cas9 system, which belongs to Class 2 (type II), involves the association of crRNA with a single unit of Cas protein (Cas9) for its function [26]. This system, which can potentially edit any gene or genomic region, is widely used, including in virology. The functional complex comprises Cas9 and a single-guide RNA (sgRNA); TracrRNA and crRNA can be fused into a sgRNA. According to recent studies, class 2 type VI CRISPR effector Cas13 can efficiently target and cleave RNA instead of DNA in different model systems, including mammalian cells [27, 28]. Thus, the CRISPR-Cas13 system offers the potential to detect RNA viruses and treat RNA virus infections. Cas13 proteins have been classified into several types, each containing two higher eukaryotes and prokaryotes’ nucleotide (HEPN)-binding domains necessary for RNA degradation [29–31]. Additionally, Cas13 does not require a protospacer adjacent motif (PAM) sequence, increasing the flexibility of crRNA target sites [32].

While CRISPR/CAS system promises great advances in the genomic and proteomic modification, it still has its own limitations. In spite of being highly specific in-silico, CRISPR/Cas system can show multiple off-target properties, potentially compromising the expected results, especially in-vivo [33, 34]. Furthermore, there have been reports indicating that human cases can develop anti-Cas antibodies, which in turn can limit the use of the CRISPR/Cas pathway in the antibody-positive cases [35].

## Loss-of-function and gain-of-function CRISPR screens

**Fig. 1:**
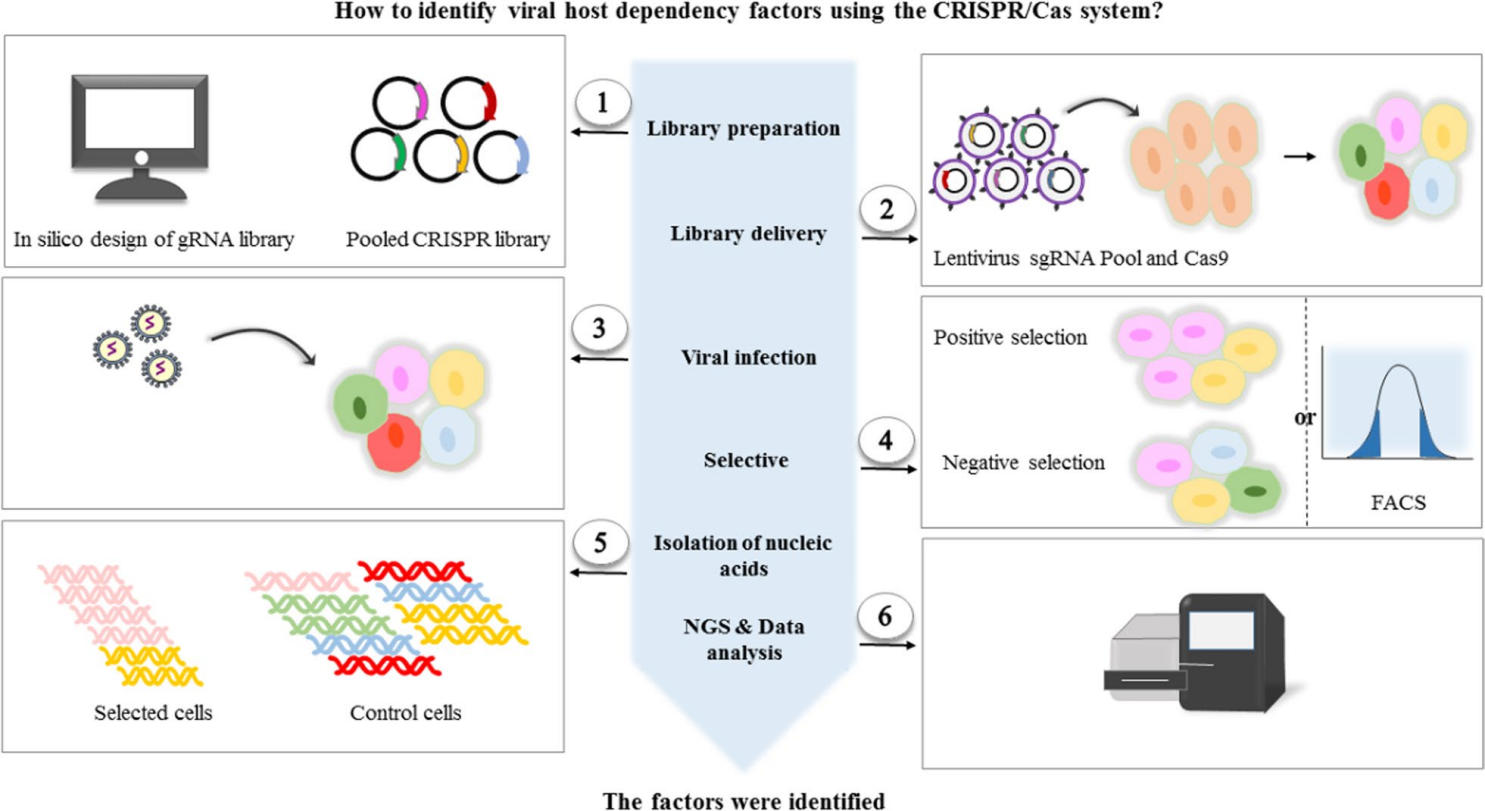
Schematic workflow diagram of CRISPR/Cas9 screen. (1) In the first step, the guide RNAs are either designed in silico or pre-made gRNA libraries are used; then cloning and validation of the gRNA library begins. (2) Packaging the gRNAs into lentiviruses and transducing the target cells with these lentiviruses, which disrupts or leads to gene expression. (3) The pooled mutagenized cell population is then infected with the virus. (4) After the infection, the selection is performed based on the survival or death of the host cells. If the infection occurs with a cytolytic virus, it allows the selection of virus-resistant cells (positive selection) in cell viability-based screens. In these survivors, the knockout of host factors contributing to viral pathogenesis will enable them to survive. Negative screens aim to identify cells that cannot survive the selection pressure. Therefore, it is often necessary to use negative screens to recognize essential genes which their loss of function will not contribute to the cell's survival. Alternatively, fluorescence-activated cell sorting (FACS) can be utilized to investigate persistent or non-cytolytic viruses. (5) Genomic DNA extraction of the selected cells and control cells is conducted, and then PCR amplification occurs. (6) Genes that are enriched or depleted in comparison to the control population are determined using Next-Generation Sequencing (NGS) analysis

To identify host factors promoting virus replication, we can perform the loss-of-function (LOF) approach in the permissive host cells and the gain-of-function approach in the non-permissive host cells. By contrast, gain-of-function and loss-of-function strategies are also used in permissive and non-permissive host cells to determine suppressor factors, respectively [36]. We have different CRISPR screens depending on their mechanism of action: CRISPR activation (CRISPRa), CRISPR interference (CRISPRi), and CRISPR knockout (CRISPR-ko) screens. CRISPRi and CRISPR-ko are loss-of-function approaches offering unprecedented opportunities to discover genes (Fig. 1). Before CRISPR was established, insertional mutagenesis in haploid stem cells and RNAi were used to edit genes, providing significant insights into understanding the host factors critical to viral replication and inhibition; nevertheless, the implementation of these techniques is restricted by having serious off-target effects [37]. Furthermore, the loss of function screens cannot reveal all the relevant pathway factors; each screen is conducted in a particular cell line, which may lack the expression or redundancy of certain relevant elements. Gain-of-function screens can overcome these limitations. CRISPRa screening is a gain-of-function approach which uses modified Cas9 protein as a transcription factor [38]. H840A and D10A mutations at HNH and RuvC endonuclease domains create a nuclease, ‘dead’ Cas9 (dCas9) molecule, revoking DNA cleavage, but is still capable of interacting with sgRNA and can bind to targeted DNA sites [39]. dCas9 can fuse to gene activation domains (VPR [40], SunTag [41, 42], SAM [43]), activating gene expression. Notably, CRISPRa is a convenient alternative which allows host-virus geneticists to conduct thorough, potent, and accurate gain-of-function studies compared to cDNA overexpression systems that usually overlook functional gene isoforms [43].

Even though the CRISPR/Cas system can be a very useful method in tracking cellular pathways through loss-of-function and gain-of function studies, the application of its results in the complex organisms and especially in the human body should be done with great caution. Gain-of-function approaches might lead to unwanted activation of potentially harmful pathways, leading to toxic end-products; while loss-of-function methods, through off-target functions, might inhibit vital cellular pathways, leading to cellular damage [33]. These events also happen in-silico and in-vitro, which might lead to a misunderstanding of the studied pathways. Furthermore, any “hit” by the CRISPR screens is considered to be valid, and concepts such as false-negative or false-positive have no place in the interpretation of the screens; this, although makes the CRISPR system specific, it can also make the results difficult to analyze. Thus, this system should be designed as specific to the desired target as possible to avoid any kind of the aforementioned side effects. It should be noted that any identified gene roles in CRISPR studies need to be further confirmed by other experiments outside the context of CRISPR screens; this necessitates follow-up experiments to be carried out via multiple other methods.

## Using CRISPR screens to understand Flaviviridae pathogenesis

Since human pathogenic viruses exploit host factors, investigations are currently exploring the host functions leading to the viral infection. The CRISPR genetic screening strategy has successfully recognized host factors essential for entry, replication, and the spread of viruses [15]. After the Flaviviruses bind to the receptors of the host cell [44] and subsequent clathrin-mediated endocytosis occurs, the viral genome is finally released to the cytosol [45–47]. The viral RNA by its 5′-cap structure binds to ribosomes, and a viral polyprotein is produced in the translation process anchoring to the endoplasmic reticulum (ER) membrane. The viral polyprotein cleavage by the viral and cellular proteases results in the structural and non-structural proteins of the virus [44, 48]. Remarkably, other viral life cycle processes, such as the viral RNA replication and virion assembly, occur at the ER site. However, despite the awareness about these processes, there is little in-depth knowledge of the involved host proteins. According to the investigations performed so far, CRISPR screens indicated that flaviviruses require ER protein complexes for their replication cycle. These screens have recognized some of the ER proteins needed for the viral replication cycle. Besides, host factors with strong activity against various viruses can also be recognized through CRISPR-mediated screenings. In this regard, many libraries exist for studying particular sets of genes, such as pooled sgRNA libraries for investigating interferon-stimulated genes (ISGs) [49]. In the following sections, we have discussed these factors thoroughly. Host factors involved in viral replication, as well as novel antiviral genes identified using the CRISPR screens, are listed in Tables 1 and 2, respectively.

**Table 1:** Proviral host factors identified utilizing CRISPR screens

Type of genetic screen	Virus	Cell line	Top screening hits	References
CRISPR KO	DENV	Huh7.5.1	OST complex (STT3A and STT3B), TRAP complex (SSR1, SSR2, SSR3), ERAD (SEL1L, AUP1, DERL2)	[14]
		Huh7.5.1	MAGT1	[50]
		Huh7.5.1, HEK293FT	EMC	[51]
		HAP1	DPM1 and DPM3	[52]
		HAP1	RACK1	[53]
	ZIKV	H1-HeLa	EMC, AXL, STT3A, TRAP complex	[54]
		HAP1	TMEM41B and VMP1	[55]
		Huh7.5	RACK1	[56]
	WNV	293FT	EMC2, EMC3, SEL1L, DERL2, UBE2G2, UBE2J1, HRD1	[57]
		293T	STTA3, SPCS1, SPCS3, SEC63	[58]
	YFV	HAP1	TMEM41B and VMP1	[55]
	HCV	Huh7.5	CD81, OCLN, CLDN1	[59, 60]
		Huh7.5.1	ELAVL1, RFK, FLAD1	[14]
CRISPRi	HCV	Huh7.5	TRIM26	[61]
CRISPRa	ZIKV	A549,SNB-19	RhoV, WWTR1	[62]

*CRISPR* clustered regularly interspaced short palindromic repeats, *CRISPRi* CRISPR interference, *CRSPRa* CRISPR activation, *CRISPR-KO* CRISPR knockout, *DENV* dengue virus, *ZIKV* zika virus, *YFV* yellow fever virus; *WNV* West Nile virus, *HCV* hepatitis C virus, *OST* Oligosaccharyltransferase, *TRAP* translocon-associated protein, *ERAD* endoplasmic reticulum-associated degradation, *MAGT* magnesium transporter, *EMC* ER membrane complex, *DPM* dolichol-phosphate mannose, *RACK1* receptor for activated protein C kinase 1, *TMEM41B* transmembrane protein 41B, *VMP1* vacuole membrane protein 1, *SPC* signal peptidase complex, *UBE2J1* ubiquitin-conjugating enzyme E2 J1, *RFK* riboflavin kinase, *OCLN* occluding, *FLAD1* flavin adenine dinucleotide synthetase 1, *TRIM26* tripartite motif containing 26, *WWTR1* WW domain containing transcription regulator 1, *RhoV* Ras homolog family member V

**Table 2:** Antiviral host factors identified by CRISPR screens

Virus	CRISPR screen	Cell type	CRISPR target genes	References
ZIKV	CRISPRa	Huh7	TMEM120A	[77]
		Huh7.5	IFI6, IFN-λ2	[79]
	CRISPR KO	Human pluripotent stem cell (hPSC)-derived neural progenitors (NPs)	ISG15, SOCS3, STAT3	[80]
		A549	RIG-I, MDA5, IFNAR	[82]
		A549	OAS3, RNAseL, STAT1, STAT2, MAVS	[85]
DENV	CRISPR KO	A549	OAS3, RNAseL, STAT1, STAT2, MAVS	[85]
WNV	CRISPR KO	A549	OAS1, OAS2, OAS3, RNAse L	[83]
YFV and other flaviviruses	CRISPR KO	Huh7.5	IFI6, HSPA5	[49]
HCV	CRISPR KO	Huh7.5	STAT1, STAT2, IRF9, STAT3, STAT6	[84]

*CRISPR* clustered regularly interspaced short palindromic repeats, *CRISPRi* CRISPR interference, *CRSPRa* CRISPR activation, *CRISPR-KO* CRISPR knockout, *DENV* dengue virus, *ZIKV* Zika virus, *YFV* yellow fever virus, *WNV* West Nile virus, *HCV* hepatitis C virus, *TMEM* transmembrane protein, *IFN* interferon, *ISG* interferon-stimulated gene, *SOCS* suppressor of cytokine signaling, *RIG* retinoic acid-inducible gene, *IFI* interferon alpha inducible protein, *MDA* melanoma differentiation-associated protein, *OAS* oligoadenylate synthetase, *MAVS* mitochondrial antiviral signaling protein, *HSPA* heat shock protein family A

## Viral entry

CRISPR Screens have displayed different cell surface molecules employed by several flaviviruses to enter the cell. Researchers have used CRISPR/Cas screens to investigate the hepatitis C virus (HCV) notably; prominent genes identified in the HCV screens were distinct from those found in other Flaviviridae members. They included genes related to viral receptors, RNA-binding proteins, and enzymes involved in metabolism [14]. It is known that various host factors such as CD81, OCLN, and CLDN1 mediate the entry of HCV into the host cells, which have been identified as critical genes through CRISPR/Cas screens [59, 60]. Based on a CRISPR/Cas9 genome-wide screen, integrin αvβ5 was found to be a ZIKV internalization factor. Significantly, the results showed that αvβ5 blocking antibody or two inhibitors, such as cilengitide and SB273005, can reduce ZIKV infection, indicating that αvβ5 integrin can act as a potential therapeutic target [63]. In another study, two important proviral factors, Ras homolog family member V (RhoV) and WW domain-containing transcription regulator 1 (WWTR1), through using CRISPRa screen were discovered. It has been demonstrated previously that WWTR1 plays a crucial role in the replication of ZIKV [64]. This study focused on the role of the RhoV factor and found that it plays a crucial role in the viral infection process of several flaviviruses, particularly the ZIKV. It seems that RhoV probably acts through its GTPase activity and its downstream effector protein, Pak1, at the step of endosomal entry, and thus increases the viral infectivity. This finding is considered to be significant since RhoV acts in the early stages of the viral cycle and can be a promising target for antiviral therapy [62].

## Viral replication

### OST complex

The N-linked glycosylation of newly synthesized proteins is catalyzed by the ER-associated oligosaccharyltransferase (OST) complex. The two distinct OST multiprotein complexes comprise a catalytic subunit (STT3A or STT3B) and other accessory subunits in mammalian cells [65]. Several studies on flavivirus screens have revealed the association of one or more OST components with flavivirus infections. Notably, this association was identified with viral RNA synthesis but not for other steps of the viral replication cycle, such as viral entry or translation. Marceau et al. demonstrated that the OST complex was associated with flavivirus replication by binding to viral non-structural proteins, which then form the RNA synthesis complex at the ER. Moreover, they identified that DENV required both STT3A and STT3B isoforms for replication, and the knockout of either isoforms completely abrogated DENV replication. However, only the STT3A knockout affected the replication of WNV, YFV, and ZIKV. This was achieved while they discovered that the catalytic N-glycosylation activity of the OST complexes was dispensable for DENV replication. Furthermore, when they utilized the catalytic mutants of STT3A and STT3B, they found that the catalytically inactive mutant proteins could support DENV propagation in the knockout cell groups. It can be concluded that the aforementioned data indicate the structural function of OST in viral replication pathway [14]. Overall, they demonstrated that canonical OST activity is dispensable for DENV replication; yet, OST complexes act as scaffolds for DENV replication. On the other hand, Lin et al. also studied other aspects of the OST complex function in DENV infection, and found that the STT3B-containing OST, through the MAGT1 subunit, is necessary for DENV propagation. Remarkably, they showed that the oxidoreductase activity of the OST MAGT1 subunit is necessary for DENV amplification, suggesting that the OST complex provides oxidoreductase activity through the OST subunit MAGT1 [50].

### ERAD pathway

Another critical factor in the flavivirus replication cycle reported by several studies, is the components of the ERAD pathway [14]. This pathway provides a protein quality control mechanism in the ER lumen [66]. CRISPR/Cas screens revealed two categories of ERAD components; first, classical ERAD machinery components, including derlin 2 (DERL2), SEL1L, and ubiquitin-conjugating enzyme E2 J1 (UBE2J1) [67]; and second, components of the ER membrane complex (EMC) [66]. The mosquito-borne flaviviruses, including DENV, ZIKV, and WNV, were found to be dependent on the EMC for the expression of their viral polyproteins. Specifically, the EMC engages with transmembrane domains (TMDs) in NS4A and NS4B during the translation process to ensure accurate topology, correct folding, and stable expression. Ngo et al. observed a notable decrease in viral RNA, especially for WNV, ZIKV, and DENV, but not for HCV, after EMC4 knockout [51]. According to a study by Ma et al., the knockout of several genes, including EMC2, EMC3, SEL1L, DERL2, UBE2G2, UBE2J1, and HRD1, inhibited WNV-induced cell death but did not affect WNV replication. These seven genes of the ERAD pathway connect WNV replication to downstream cell death pathway(s) [57]. Neuronal cell death is one of the most common causes of death due to WNV infection, and these proteins might also serve as new therapeutic targets. Furthermore, another study found that ZIKV, DENV, and YFV strongly required EMC for replication in the early stages of infection [54]. The results of these studies show distinct roles for EMC in the flavivirus infection cycle. The results first emphasize the effective role of EMC in replicating DENV, ZIKV, and YFV viruses. They then describe its function, which is required for WNV-induced cytopathic role (cytopathicity). However, the exact mechanism through which EMC acts in flavivirus infections should be examined more broadly.

### TMEM41B and VMP1 proteins

**Fig. 2:**
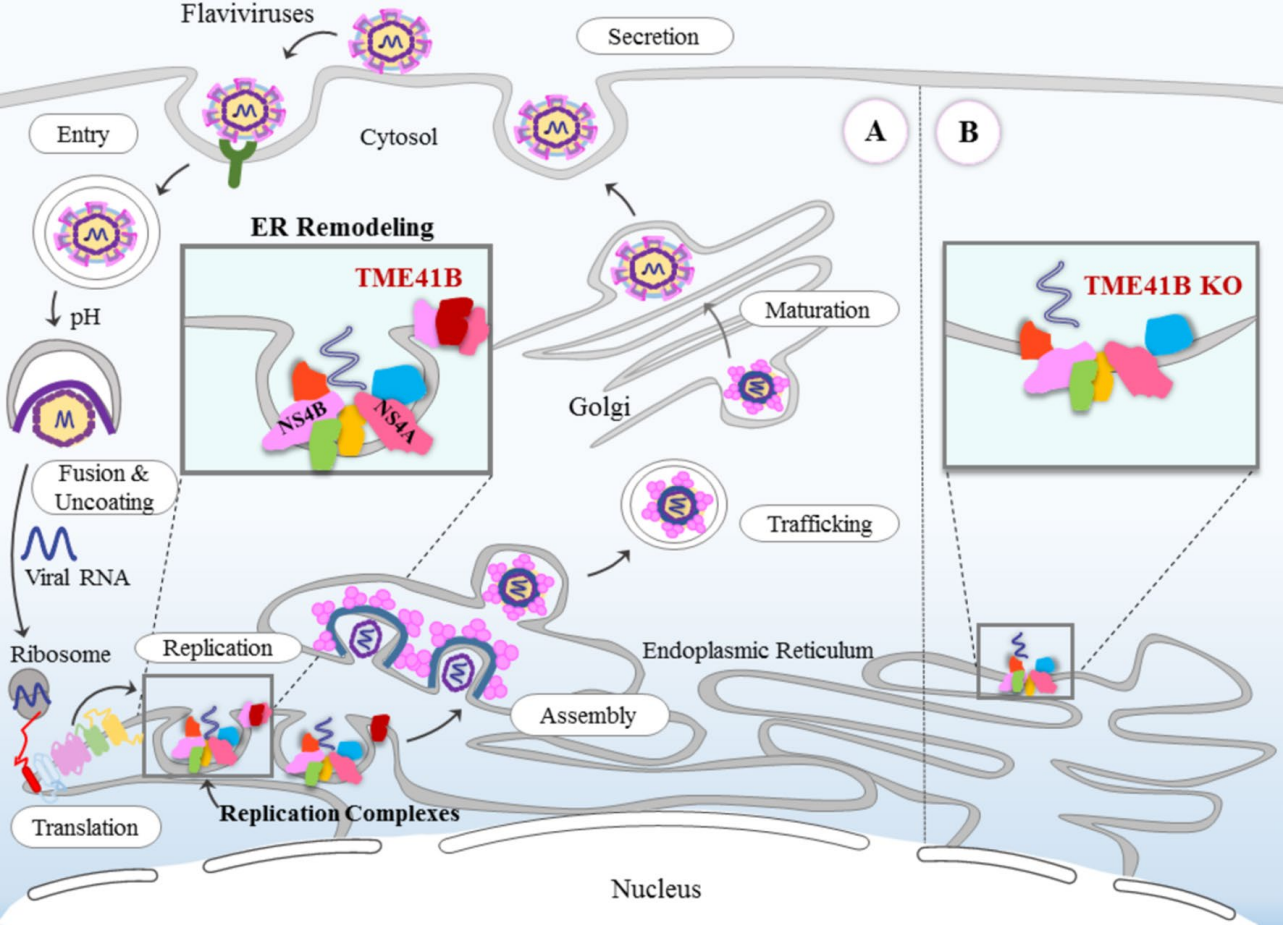
Flavivirus replication cycle and the role of TMEM41B as an ER remodeling protein. A: wild‐type cells; B: TME41B KO cells

Transmembrane protein 41B (TMEM41B) is a multi-spanning membrane protein found in the endoplasmic reticulum (ER). TMEM41B has similar roles to vacuole membrane protein 1 (VMP1), such as autophagy and lipid mobilization [68–70]. The CRISPR/Cas9 screens have recently revealed that TMEM41B is a pan-flavivirus host factor; it has also been identified that in addition to ZIKV, YFV, HCV, WNV, and DENV-2, several other members of the Flaviviridae family require TMEM41B for causing infection. TMEM41B and VMP1 might also be involved in remodeling cell membranes through their association with flavivirus proteins. Indeed, flaviviruses may hijack these proteins for their ability to remodel host cell membranes that are required to form viral replication compartments in the ER. In particular, Hoffmann et al. have suggested that together with NS4A and NS4B, TMEM41B is recruited at the ER membrane where replication complexes are formed, then TME41B reduces the local free energy imposed by the NS4A- and NS4B-induced membrane curvatures (Fig. 2) [55].

### DPMS complex

Utilizing a genome-wide CRISPR/Cas9 screen, Athena et al. identified two resident subunits of the endoplasmic reticulum incorporating dolichol-phosphate mannose synthase (DPMS) complex, DPM1 and DPM3, and the role they play in DENV infection. They discovered that DPM1 or DPM 3 are required for the efficient infection by all the DENV serotypes. Moreover, ZIKV and YFV 17D infection was remarkably inhibited in cells lacking DPM1 or DPM3. According to this study, DENV requires DPMS to regulate viral RNA replication and proper glycosylation of the viral proteins E, prM, and NS1 [52].

### RACK1

The Receptor for Activated Protein C Kinase 1 (RACK1) is a core component of the 40S ribosomal subunit and has a significant role in several aspects of cellular functions [71, 72]. Recently, according to a CRISPR/Cas9 KO screen, RACK1 was identified as a novel host factor that seems to be required for ZIKV replication. The function of this factor is crucial for viral RNA genome replication. In particular, NS1 is essential for the biogenesis of viral replication factories known as ‘vesicle packets’ (VPs), and RACK1, through interaction with NS1 within the ER lumen, playing a key role in the construction of replication organelles early in the virus lifecycle. As a result, RACK1 depletion leads to changes in morphology and decreases the frequency of VPs [56]. In addition, another study examined the function of RACK1 during the life cycle of DENV. RACK1, in association with Vigilin and SERBP1 factors which interact with DENV viral RNA, forms a ternary complex mediating viral replication [53].

### TRIM26

**Fig. 3:**
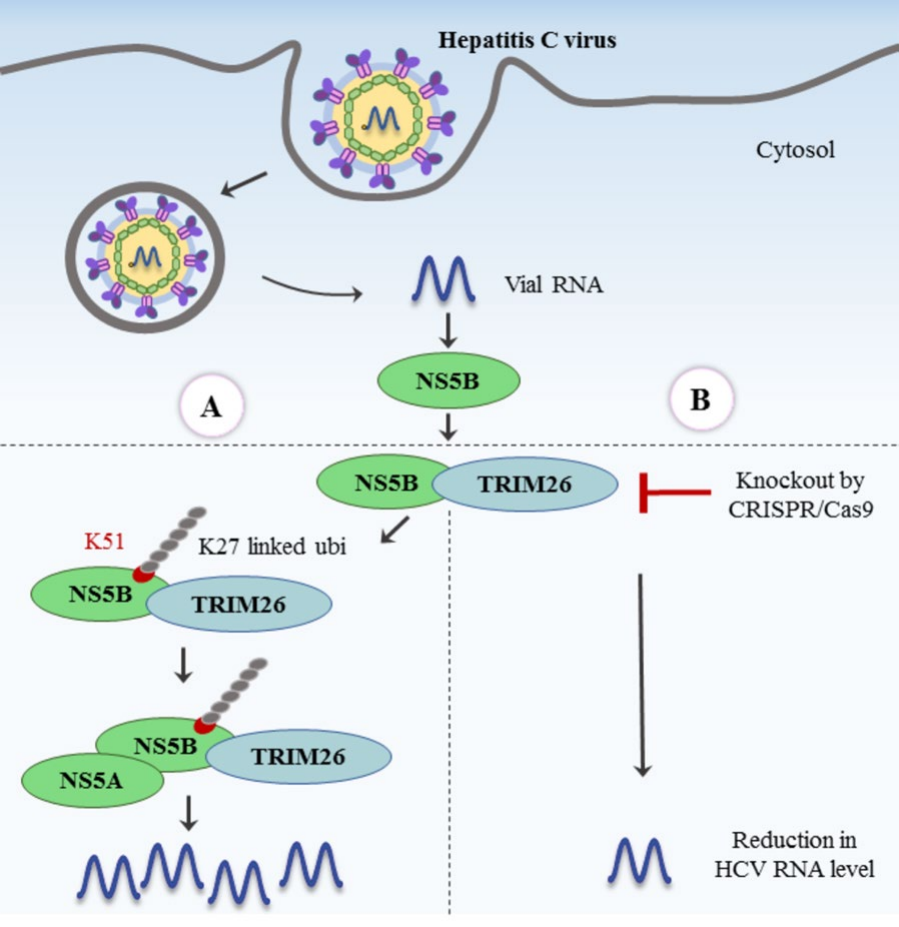
The role of TRIM26 in promoting HCV genome replication. A: wild‐type cells; B: TRIM26 knockout cells

The host factors involved in HCV replication are also of tremendous importance. Liang et al., using a CRISPR/Cas9 screen, showed that TRIM26, an E3 ubiquitination ligase, is a critical host factor for HCV (Fig. 3). They revealed that TRIM26 interacts with the NS5B protein and thus mediates its K27-linked ubiquitination at residue K51, increasing NS5B-NS5A interaction. The knockout of TRIM26 significantly diminished HCV replication, but did not terminate it entirely. In contrast, TRIM26 seems to play a virus-specific role in HCV replication since it is not involved in DENV and ZIKV life cycles [61].

### ELAVL1, RFK, and FLAD1

One of the RNA-binding proteins involved in mRNA stabilization is ELAVL1; since it has been observed that HCV RNA replication in the ELAVL1-knockout cells was significantly reduced. This suggests that ELAVL1 is crucial for the HCV RNA replication. HCV screens have also contributed to the discovery of the role of the enzymes involved in the HCV replication. For example, riboflavin kinase (RFK) and FAD synthase (FLAD1) knockout cells, two enzymes involved in the conversion of riboflavin (vitamin B2) to FAD, were resistant to HCV replication. This is while FAD rescued HCV replication in these knockout cells; therefore, a link between intracellular FAD levels and HCV RNA replication was discovered. Hence, Lumiflavin, which is an inhibitor of the cellular uptake of riboflavin, can inhibit viral RNA replication [14].

## Polyprotein processing and viral translation

The signal peptidase complex (SPC) is a membrane complex in the endoplasmic reticulum, where it cleaves signal peptides (SPs) from the N-termini of the secretory and membrane proteins. A subset of signal peptidase complex (SPCS) proteins is required for efficient cleavage of flavivirus structural proteins (prM and E) and the secretion of viral particles; based on the CRISPR/Cas9 screen, Knockout of this factor indicated intense defects in the polyprotein cleavage of several Flaviviridae family members [58]. Another known factor is the translocon-associated protein (TRAP) complex (containing subunits SSR1, SSR2, and SSR3), which facilitates the translocation of proteins across the endoplasmic reticulum membrane [73]. The TRAP complex plays a crucial role in DENV-2, ZIKV, and YFV RNA replication [14]. A recent study has shown that host factors SBDS and SPATA5, which are involved in the formation of ribosomes, are required for the synthesis of viral proteins. Losing these 60S ribosome biogenesis proteins leads to a decrease in the viral replication of flaviviruses and several other viral families. The study specifically found that the loss of function of these two proteins, which are essential for the formation of the 60S ribosome part, causes defects in the processing of rRNAs and the assembly of ribosomes [74].

## Anti-viral immunity

### Host antiviral restriction factors

**Fig. 4:**
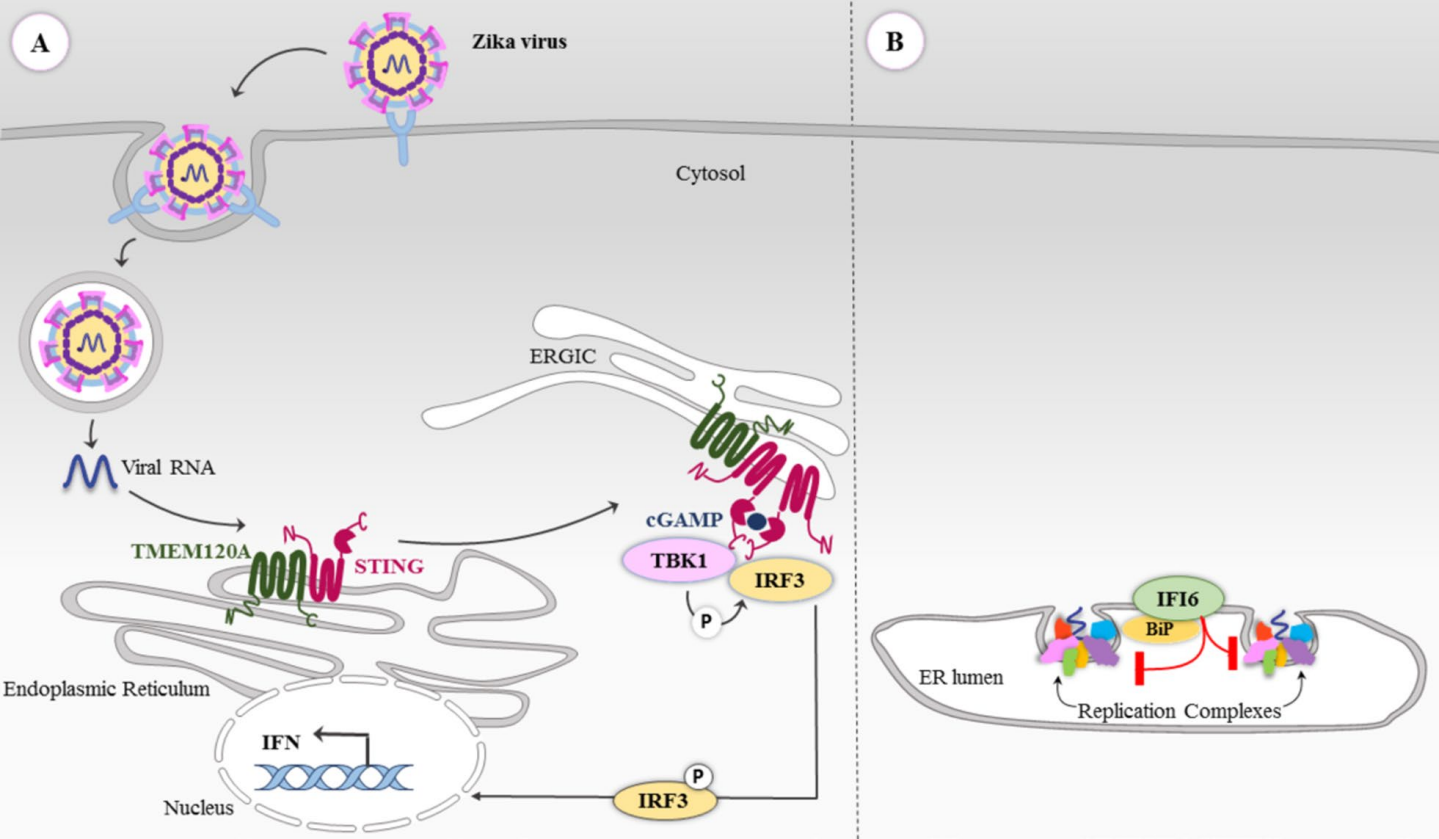
**A** Schematic of the antiviral function of TMEM120A. **B** The antiviral role of IFI6 that inhibits the formation of a replication organelle through interaction with BiP

Although many host proteins are hijacked or disrupted by viral infections, various studies have shown that several host proteins operate with antiviral properties acting against viral infection. In this regard, a gain-of-function screen has introduced TMEM120A as a host restricting factor against ZIKV infection. TMEM120A is a transmembrane protein localized on the plasma membrane, nuclear membrane, and endoplasmic reticulum [75–77]. TMEM120A displays an antiviral function through interaction with STING; it promotes STING translocation from the endoplasmic reticulum to the ER-Golgi intermediate compartment (ERGIC), leading to the activation of TBK1 and phosphorylation of the transcription factor IRF3. Subsequently, it results in the activation of type-I interferon (IFN) expression (Fig. 4A) [77]. On the other hand, CRISPR screens have discovered interferon responses to flaviviruses. The IFN response can target viral replication at the ER. For instance, IFI6 (encoding Interferon alpha-inducible protein 6) and HSPA5 (encoding endoplasmic reticulum chaperone BiP) are two genes involved in this antiviral mechanism. IFI6, localized to the ER and stabilized by its interaction with BiP, prevents the formation of virus-induced ER membrane invaginations, hence suppressing the viral life cycle (Fig. 4B) [49]. Moreover, a CRISPR activation screen recognized that IFI6 and other ISGs, including Interferon Lambda 2 (IFN-λ2), can rescue cells from ZIKV infection. On the other hand, it has recently been found that ZIKV can evade the BiP/HSPA5 pathway by down regulating their expression (78). The aforementioned point alongside the other mostly controversial roles suggested about the mechanisms of this pathway, necessitates further studies to be conducted to clarify whether the BiP/HSPA5 can lead to novel antiviral therapies. Collectively, these studies show the potential of various CRISPR screening strategies in recognizing host factors that either facilitate or inhibit viral replication [79].

### Regulation of interferon signaling pathways

CRISPR screening allows researchers to identify genes regulating interferon (IFN) responses to flavivirus infections. According to the investigations, ZIKV causes proliferation loss and cell death in human neural progenitors (NPs) during early cortical development, resulting in fetal brain abnormality. In this regard, a study identified host genes in human NPs associated with ZIKV infection, such as host factors involved in the interferon activity. The results revealed that knocking out IFN pathway regulators in NPs, including ISG15 and SOCS3, reduced the infectivity of ZIKV. Therefore, ZIKV seems to evade the host antiviral defense by relying on negative regulators of the IFN pathway [80].

Viral RNAs are identified by RIG-I-like receptors (RLRs), including RIG-I and MDA5, leading to the activation of RIG-I/MAVS, a significant immune pathway [81]. Therefore, inactivating RIG-I impairs the response against these pathogens. CRISPR-KO demonstrated that RIG-I, but not MDA5, was the major sensor for the recognition of ZIKV RNA in A549 cells. When RIG-I recognizes ZIKV infection, it ultimately leads to the induction of type I IFNs and ISGs. The lack of RIG-I causes ISG expression to diminish in the ZIKV-infected cells, increasing the viral replication and apoptosis [82]. RNase L, an antiviral enzyme with several functions such as the degradation of viral and cellular RNAs, inhibiting protein synthesis, and restricting the replication and spread of various viruses, is activated by the 2′,5′ -oligoadenylates after the infection with RNA viruses. It should be noted that different oligoadenylate synthetases (OAS), OAS1, OAS2, and OAS3, synthesize 2′,5′-oligoadenylates. Using CRISPR-KO, it was uncovered that in this process, OAS3, but not OAS1 or OAS2, is a major factor involved in 2′,5′-oligoadenylate synthesis for RNAse L activation. This study also revealed that in RNase L- and OAS3-KO cells, the replication of four viruses, including WNV, was increased. According to these results, OAS3 may serve as an antiviral target, while OAS1 and OAS2 may have alternative functions [83]. Studies have been conducted to confirm and characterize the main components of IFN pathways using the CRISPR system. A study explored the function of STAT1 and STAT2 in inhibiting HCV replication through IFN-α and IFN-λ. STAT1 and STAT2 play a role in the early induction of ISGs in response to IFN-α in Huh-7.5 cells. However, IFN-α can somewhat inhibit HCV replication in the absence of STAT1. It seems that this inhibition is mediated by STAT2 and IRF9, but not STAT3 or STAT6. Meanwhile, IFN-λ inhibits HCV replication only through a STAT1-dependent pathway; while STAT1 inactivation fully suppresses the IFN-λ antiviral activity. According to the results, the induction of ISGs, like PKR and IRF9, by IFN-λ was inhibited by the knockout of STAT1 in HCV-infected cells [84].

### Viral evasion

Viruses use immune evasion mechanisms to increase replication and counter host immune surveillance. CRISPR/Cas has been used to study several of such mechanisms. For instance, to investigate the effects of RNase L on ZIKV infection, CRISPR-KO was used to knockout targeted host genes involved in the RNase L pathway. The results demonstrated that ZIKV genome was decreased in the infected wild-type cells compared to RNase L KO cells; while the amount of infectious ZIKV released from the wild-type cells were notably higher than the RNase L KO cells. According to investigations, it seems that ZIKV can escape cleavage by RNAse L due to the formation of replication factories in the membrane of the ER. Therefore, ZIKV genomes resist RNAse L cleavage in such replication factories. While DENV generates replication factories, it is not resistant to RNAse L-mediated cleavage. Consequently, it can be said that this mechanism is specific to ZIKV within the flaviviruses [85]. Studies have identified the interaction of an inactive form of RNase L with actin cytoskeleton to reorganize cellular framework during viral infection [86]. Accordingly, in a recent study, they investigated the role of RNase L during Zika virus infection, and the results indicated the proviral role of inactive RNase L during ZIKV infection. ZIKV induces cytoskeletal remodeling during infection to form replication factories (RFs); the absence of RNase L results in defective remodeling of microtubules. In general, it can be concluded that ZIKV exploits the interaction between RNase L and the cytoskeleton to facilitate ER rearrangement to create RFs, promoting ZIKV production [87].

## CRISPR/Cas antiviral strategies against Flaviviridae viruses

CRISPR/Cas technology is a growing field in the prevention and treatment of viral infections. The outbreaks of Flaviviridae members in different parts of the world in recent years emphasize the need for innovative methods of vector control which is a strategy used to limit the transmission rate of these viruses. Furthermore, CRISPR/Cas tools are being used to generate gene drives that can potentially decrease mosquito populations. The use of gene drives for mosquito control has attracted much attention in the recent years, as the ease of production of CRISPR-based gene drive systems sets them apart from other methods [88, 89]. There have been multiple methods introduced in CRISPR-based gene drive systems, which include: suppression drives, through which a weakening gene is inflicted in a target population, limiting their activity or even eradicating the targets; and Modification drives, by which the target population is altered in a desired way (e.g. in the setting of malaria control the mosquitoes are altered not to be capable of transmitting the disease) [90].

### CRISPR/Cas9

In general, CRISPR-based antiviral approaches include the inactivation of genes involved in the progression of viral infections and include two modes of knock‐out or knock‐down of viral genes or relevant host factors. Some fundamental challenges in using this system include the following: There is a possibility of off-target mutagenesis, and Cas9/gRNA expression level and duration are also significant factors. Delivering the CRISPR/Cas9 system in vivo efficiently and safely is a considerable clinical challenge [91, 92]. One of the other challenges is the human body’s immunogenicity against the Cas9 protein, which is derived from bacteria [93]. Another important point in CRISPR-Cas9-based therapeutic is related to genome repair or rearrangement processes after double-stranded breaks, which may lead to unexpected mutations [94]. On the other hand, the canonical CRISPR/Cas9 is unable to perform its editing function for the RNA virus genome. The Cpf1 and C2c2/Cas13 among all CRISPR/Cas systems are the ones able to be designed to target the RNA virus genome [95]. The Cas9 endonuclease from Francisella novicida (FnCas9) has also been reported to target endogenously transcribed mRNA and thus regulate gene expression (96). Today, most HCV infections can be treated with appropriate pharmacological interventions. Nevertheless, we may face drug-resistant mutant HCV variants, so the current anti-HCV regimen may not be effective in the future. Therefore, the CRISPR/Cas9-mediated disruption of the HCV genome may be suggested as an anti-HCV strategy. In a study using FnCas9, the HCV RNA genome was targeted in eukaryotic cells, which resulted in the inhibition of viral protein production. In fact, by targeting the 5′-and 3′-UTR of the HCV genomic + ssRNA, virus inhibition was observed due to the blockade of viral RNA translation and viral replication machineries [97].

### CRISPR/Cas13

The application of the CRISPR/Cas13a (known previously as C2c2) system to RNA editing has expanded [27, 98]. For example, in a study, Cas13a was reported to target HCV internal ribosomal entry site (IRES), reducing the HCV RNA replication and translation. Thus, using IRES-specific crRNAs, Cas13a can suppress HCV more efficiently in huh-7.5 cells [99]. Moreover, Li et al. [100] hypothesized that the CRISPR/Cas13a system could suppress DENV infection by degrading viral RNA genome or by mutagenizing crucial genomic elements; therefore, they adapted the CRISPR-Cas13a system to DENV and discovered a CRISPR RNA (crRNA) that was able to suppress DENV replication in the cell culture system by targeting the NS3 gene. Another study used a novel strategy using CRISPR/Cas13 against RNA viruses. They indicated that virus-like particles (VLP) could be used to deliver PspCas13b RNP to primary human target cells to suppress dengue virus infection effectively. Shortening the spacer length of crRNA in the range of 18–26 nts improved CRISPR/Cas13b knockdown activity without compromising crRNA processing or multiplex targeting capability [101]. On the other hand, Chen et al. aimed to develop an anti-ZIKV system using CRISPR/Cas13b in mammalian cells. They first generated a cell line susceptible to ZIKV infection and a reporter system, then designed fourteen crRNAs, five of which were effective in targeting conserved regions of the ZIKV genome. Such studies, which try to develop new methods of inhibiting RNA viruses, increase the hopes of using the CRISPR/Cas13 system as a new therapeutic approach in the near future [102]. The CRISPR/Cas13b technology offers tempting advantages for therapeutic purposes. Cas13b can target multiple sites at once, which significantly lowers the chance of viruses escaping the immune system. Instead of exploring the biological characteristics of viruses which are required to produce traditional antiviral drugs, crRNA can be designed just by comprehending the virus genome sequence. CRISPR/Cas13b target-cleavage of RNA is a safer alternative since it is not permanently inherited [103].

## Conclusion

Recently, CRISPR/Cas9 has revolutionized the study of host-virus biology. CRISPR/Cas technology is utilized to improve our knowledge of how viruses exploit their hosts, as well as to develop new antiviral therapies. Particularly, further studies are constantly conducted using this approach to improve our understanding of flavivirus life cycles, leading to the discovery of essential factors. However, there are still numerous obstacles that need to be addressed. Viral host factor requirements may differ based on viral strains and cell types. In this regard, studies investigating host-virus interactions using CRISPR must use clinically and epidemiologically important virus isolates and corroborate the results with numerous strains. It is also possible that host factors vary from one cell type to another; for example, ZIKV, which infects specialized cells like neural stem cells. Therefore, CRISPR screens should be conducted in the proper cell contexts to reveal the involved factors comprehensively [104]]. Undoubtedly, future screens will provide insight into how viruses have evolved to exploit and subvert host functions. In addition, the findings may lead to potential targets for antiviral therapy. Finally, we expect that next generation CRISPR approaches alongside other new technologies will help us better understand complex biological processes.

## Data Availability

Not applicable.

## Abbreviations

CRISPR: Clustered regularly interspaced short palindromic repeats
CRISPRa: CRISPR activation
CRISPRi: CRISPR interference
CRISPR-KO: CRISPR knockout
dCas9: Dead Cas9
DENV: Dengue virus
DPMS: Dolichol-phosphate mannose synthase
EMC: ER membrane complex
`ER: Endoplasmic reticulum
ERAD: Endoplasmic-reticulum-associated degradation
ERGIC: ER-Golgi intermediate compartment
FACS: Fluorescence-activated cell sorting
FLAD1: Flavin adenine dinucleotide synthetase 1
HCV: Hepatitis C virus
IFN: Interferon response
IFN-λ2: Interferon lambda 2
ISGs: Interferon-stimulated genes
JEV: Japanese encephalitis virus
MAGT: Magnesium transporter
NGS: Next-generation sequencing
NPs: Neural progenitors
OAS: Oligoadenylate synthetases
OST: Oligosaccharyltransferase
RACK1: Receptor for activated protein C kinase 1
RFK: Riboflavin kinase
RLRs: RIG-I-like receptors
RNAi: RNA interference
RhoV: Ras homolog family member V
sgRNA: Single-guide RNA
SPC: Signal peptidase complex
SPs: Signal peptides
STAT: Signal transducer and activator of transcription
TBEV: Tick-borne encephalitis virus
TMDs: Transmembrane domains
TMEM41B: Transmembrane protein 41B
TRAP: Translocon-associated protein
TRIM26: Tripartite motif containing 26
UBE2J1: Ubiquitin-conjugating enzyme E2 J1
VLP: Virus-like particles
VMP1: Vacuole membrane protein 1
VPs: Vesicle packets
WNV: West Nile virus
WWTR1: WW domain containing transcription regulator 1
YFV: Yellow fever virus
ZIKV: Zika virus
